# Evaluation of the efficacy of epiduroscopic adhesiolysis in failed back surgery syndrome

**DOI:** 10.3906/sag-1807-173

**Published:** 2019-02-11

**Authors:** Ayşegül CEYLAN, İbrahim AŞIK, Güngör Enver ÖZGENCİL, Burak ERKEN

**Affiliations:** 1 Department of Anesthesiology and Reanimation, Gülhane Education and Research Hospital, Ankara Turkey; 2 Department of Anesthesiology and Reanimation, Ankara University Faculty of Medicine Hospital, Ankara Turkey

**Keywords:** Failed back surgery syndrome, epiduroscopic adhesiolysis, hyaluronidase

## Abstract

**Background/aim:**

We aimed to compare the results of the treatment of the patients with failed back surgery syndrome (FBSS) by mechanical lysis and steroid hylase injection via epiduroscopy due to their stabilization status and to detect the effect of pathological diagnostic markers on prognosis and ongoing treatment protocol.

**Materials and methods:**

Eighty-two patients with FBSS symptoms were included. Two groups were composed as group I (stabilized) and group II (nonstabilized). All patients were evaluated using the oswestry disability index (ODI) and visual analogue scale (VAS) scores before and after treatment at 1, 3, 6, and 12 months and using the patient satisfaction scale at 12 months following treatment. Epidural scar tissue visual and mechanical signs were also recorded.

**Results:**

Mean VAS scores were 7.8 and 3.28 points in group I (P < 0.001) and 7.51 and 2.74 points in group II (P < 0.001) at the beginning and at 12 months, respectively. Mean ODI scores were 34.05 and 22.16 points in group I (P < 0.001) and 30.74 and 19.46 points in group II (P < 0.001) at the beginning and at 12 months. VAS and ODI scores decreased significantly in both groups, but were more significant in the nonstabilized group (P < 0.001). Moderate or severe fibrous tissue was observed in 86.58% of the patients and patient satisfaction scores were very good or good in 78.06% of the patients. During the procedure, a dura rupture developed in four patients in the stabilization group and in two patients in the nonstabilization group; however, none of these patients developed a spinal headache and no significant permanent complication arose.

**Conclusion:**

We suggest that epidural adhesiolysis, hyaluronidase, and steroid injection in patients with FBSS chronic low back pain and/or radicular symptoms may give reliable information about the quality of life, accuracy of diagnosis, and the possible course of the present findings and may be more effective in nonstabilized patients.

## 1. Introduction 

Although there is lack of clear consensus related to failed back surgery syndrome 

(FBSS), many definitions have been provided in the literature (1). The signs and symptoms of FBSS are low back pain, radicular pain, sphincter insufficiency, restricted movement, muscle spasms, contractures, and changes in the motor and reflex functions. FBSS treatment can be difficult and ranges from conservative treatment to reoperation (2). 

By observing adhesions directly, the lysis of scar tissue can be carried out mechanically using epiduroscopy. Adhesions can, theoretically, be disintegrated, and their evaluation scores may improve. The use of hyaluronidase with steroids in the epidural space may result in greater and longer efficacy than steroids alone (3). An epiduroscopy refers to an endoscopic technique for the observation of the lumbosacral epidural interspace via a transsacral approach in patients with chronic waist pain. 

The magnification and mechanical properties of an epiduroscope allow systematic assessment using fluoroscopy, saline infusions, and the injection of radiographic agents into the epidural space (4,5). The shape of a normal epidurogram resembles a Christmas tree, on which the lack of contrast in a defined region suggests the presence of a defect (6). Normal epidural space contains white or straw-colored globular tissues of fat, duramater (gray–white in color), vessels, arteries, fibrous fibers, and ligaments. It is a wide plexus containing fibrous membranes, ligaments, lymphatic and blood vessels, and nerve tissue (6–8). 

Pathological images were evaluated in terms of color, texture, and the presence of known anatomical structures. The fibrous tissue was classified as mild, moderate, or severe according to vascular structure, resistance to device, and epidurographic images. 

Mild fibrous tissue: Increased vascularity in the epidural area and loss of fibrous fibers and layers that allows the passage of opaque material in an epidurography and that are not resistant to mechanical cleaning via the tip of the epiduroscope. 

Moderate fibrous tissue: Fibrous materials that are more regular and in continuous strands and layers, longer than mild fibrosis, and that are somewhat resistant to the advancement of the device; also, blood vessels are decreased and strands and layers of the fibrous materials partially fill the epidural space and surround nerve roots. 

Severe fibrous tissue: The scar tissue occupies a large layer over the majority of epidural area, showing great resistance to the advancement of the device and permitting a little or no movement. 

Avascular areas on the fibrous bands are very common (9). 

Epiduroscopy has some complications such as dural injury, root damage, epidural bleeding, infection, macular hemorrhage, increase in intracranial pressure (10), and symptoms of meningeal irritation or allergy due to the opaque agent (11). 

The intention of the present study was to evaluate the efficacy of epiduroscopic treatment in patients with FBSS and a history of stabilized or nonstabilized lumbar surgery in terms of raising the functional quality of life and easing chronic pain, and to evaluate the diagnostic and prognostic value of the visual data detected during the procedure. We also aimed to determine the efficacy of mechanical adhesiolysis via epiduroscopy based on the type of surgery and the relationship between the prognosis and the degree of scar tissue. 

## 2. Material and methods

Data from patients diagnosed with FBSS and underwent an epiduroscopy in the presence of waist or waist and leg pain between 2013 and 2017 were evaluated. 

All patients were evaluated using the oswestry disability index (ODI) and visual analogue scale (VAS) scores before and after treatment at 1, 3, 6, and 12 months and using the patient satisfaction scale (PSS) at the 12th month following the treatment. 

### 2.1. Patient selection 

Patients between 18 and 65 years of age with VAS scores of ≥7 points and with severe leg or low back pain and/or neuropathic findings were included in the study. All patients declared to experience pain for at least 6 months postoperatively. 

A total of 82 patients were divided into two groups as stabilized and nonstabilized, based on the type of the previous surgery. None of the patients improved after supportive treatment, physiotherapy, nonsteroidal antiinflammatory drugs (NSAIDs), or numerous treatments including steroidal or nonsteroidal sporadic epidural injections, for at least three months after surgery. None of them had used opioids, had undergone spinal cord stimulation, or had been treated with continuous psychotherapy. 

Patients with coagulopathy, glaucoma, malignancy, mental retardation, local or systemic infection, increased intracranial pressure, cerebrovascular disease, or morbid obesity were excluded from the study. 

### 2.2. Epiduruoscopy procedure

All epiduroscopic procedures were carried out by a single experienced practitioner. The patients’ blood pressures, heart rates, electrocardiography, oxygen saturation, and respiratory rates were monitored. The sacral region and the surrounding area were sterilized. All patients were administered 2 mg of midazolam and 20–50 mg of propofol to reduce anxiety. In case of pain, fentanyl 0.5 µg/kg was planned to be injected intravenously (IV). Deep sedation was avoided in order to keep the patients awake and conscious and to make a full neurological monitoring possible. In addition, 1.0 g of cefazolin was injected IV 30 min before the procedure for prophylaxis of infection. 

A local anesthetic (2–3 mL of prilocaine) was injected into the skin and subcutaneous tissues. The sacral canal was entered via the loss of resistance method and then advanced towards the cephalic direction. After injecting a nonionic radiopaque agent (iotrolan 10 mL, Isovist 240®; Schering, Osaka, Japan), a caudal epidurogram was obtained on the anteroposterior fluoroscopic image. We performed all epiduroscopies within the posterior epidural area. The Tuohy needle was removed once the guidewire had reached the desired position and the position was checked by anteroposterior and lateral fluoroscopy. After widening the guidewire entry via the dilator, a 2.6-mm (8 F) Epi-C® epiduroscopy catheter was advanced through the cannula into the epidural space using the Seldinger technique. The fluoroscopy helped to identify the level attained by the endoscopic tip. The nerve root was touched slightly with the epiduroscope and the patient was asked whether the pain was similar or equivalent to the pain experienced following the back surgery. Following adhesiolysis, 1500 IU of hyaluronidase, 100 mg of lidocaine, and 40 mg of dexamethasone were injected into the epidural space via the catheter. During the procedure, the injection was usually made within the fibrotic area where evidence of irritation was present or where the patient described the pain as being equivalent to the previous pain. The procedure was ended once the epidurogram identified the contrast media had reached the affected nerve roots. 

The epidural area was examined visually to identify any pathologic finding. When detected in the epidural space, adhesions or severe fibrotic areas were disrupted through a bolus injection of saline and the tip of the catheter was carefully and gently moved forward to achieve mechanical adhesiolysis. We did not attempt to force the epiduroscope in any direction when the width was insufficient and paresthesia or resistance was met. The fluid injection or the manipulation of the epiduroscope was performed in the direction of the area in which pain was felt. During the procedure, a standard light source and monitoring system was used (Karl Storz, Tuttlingen, Germany). Within the epidural space, physiological isotonic saline was administered in a mean volume of 100 mL in both groups, and was passively discharged by the tip of the epiduroscope. Any complications that had occurred were recorded. The procedures lasted between 30 and 45 min. 

So as to minimize direct nerve root irritation or damage during mechanical lysis, our patients were kept awake, conscious, and in communication with the surgeon. Dural ruptures may sometimes occur because of opening small holes in the dural membrane during epiduroscopy. All patients were given information preoperatively that this may lead to postdural puncture headache. The treatment is hydration, rest, and medications. Epidural blood patching was planned for patients with remaining complaints. Excessive saline infusion may suddenly and rapidly increase intracranial pressure which may cause intraorbital hemorrhage. We avoided this complication by limiting the volume of the washing liquid and by using a system to expel it from the epidural space. All patients were given antibiotics for 7 days for prophylaxis. After the procedure ended, the area was cleanly covered. The patients were discharged after 4–6 h of observation. If needed, patients with pain were given an antiinflammatory drug or paracetamol during the follow-up. They were recommended bed rest for a few days, and analgesic, antiinflammatory, and antibiotic medications. 

### 2.3. Statistical analysis 

Data analysis was carried out using the SPSS for Windows 21 package program. Descriptive statistics were expressed as mean ± standard deviation, continuous variables were expressed as median (min–max), and the number of cases and percentages (%) were used for nominal variables. A Student’s t-test and a chi-square test were used for univariate analyses. Since the expected value was below 5 and the number of cells was 50% in the contingency table, comments on the values were stated in terms of frequency and percentage, rather than the chi-square value. Within-subject effects and between-subjects effects were assessed with a mixed ANOVA. The assumption of sphericity was assessed with a Mauchly’s sphericity test. If the assumption of sphericity was not met, a multivariate ANOVA (MANOVA) was used. A Pillai’s trace test was used, as if the subjects were equal in the groups, it was robust for violation of the assumption of homogeneity of variance and the distribution of multivariate normality. Pairwise comparisons in the main effect of time were evaluated in terms of a simple contrast (first group reference). A simple effect analysis with a Bonferroni adjustment was used to resolve any significant interaction terms. A simple effects analysis was used to break down an interaction term, and clinical significance was measured with partial eta squared (effect size). The results were considered statistically significant at a P-value <0.05. A recovery rate of over 50% was considered successful (12). 

## 3. Results

### 3.1. Demographics and location of lesions

The mean age of the patients in the stabilized group was significantly higher than that in the nonstabilized group (53.09 ± 9.78 years and 48.13±10.17 years, respectively; P = 0.027). The distribution of sex in the stabilized group was similar to that in the nonstabilized group (P = 0.358). Levels of the procedure L4-L5 and L5-S1 were more frequent in the stabilized group (n = 21, 58.8%) while L5-S1 was more frequent (n = 18, 46.2%) in the nonstabilized group. The distribution of the fibrous tissue between the stabilized and nonstabilized groups was statistically different (P < 0.001) and the most common were severe fibrous tissue damage (n = 26, 60.5%) in the stabilized group and moderate fibrous tissue damage (n = 28, 71.8%) in the nonstabilized group (Table 1). 

**Table 1:** Demographic and clinical features.

		Stabilization		
		Yes, n = 43	No, n = 39	Test statistics and P-value
Age	Mean ± SD Total	53.09 ± 9.78	48.13 ± 10.17	t = 2.253* P = 0.027
		Mean ± SD = 50.73 ± 10.71		
Sex	Male (n = 44, 53.7%) Female	n = 21, 47.7%	n = 23, 52.3%	χ2 = 0.845** P = 0.358
	(n = 38, 46.3%)	n = 22, 57.9%	n = 16, 42.1%	
Fibrous tissue	Mild Moderate Severe	n = 0, %0	n = 11, 28.2%	χ2 = 39.588 P < 0.001
		n = 17, %39.5	n = 28, 71.8%	
		n = 26, %60.5	n = 0, 0%	
Lesion place	L4-L5	n = 3, 7%	n = 2, 5.1%	***
	L4-S1	n = 21, 58.8%	n = 14, 35.9%	
	L5-S1	n = 3, 7%	n = 18, 46.2%	1
	L3-L5	n = 9, 20.9%	n = 2, 5.2%	2
	L2-L3	n = 0, 0%	n = 1, 5.2%	

*Student’s t-test statistical value. **Chi-square test statistical value. ***6 cells (50.0%) have expected count less than 5.

### 3.2. VAS score results

The average VAS scores calculated at five different time points during a 12-month period were significantly different for the stabilized and nonstabilized groups. Since the interaction effect is meaningful, we prefer not to comment on the main effect of stabilization and time. The results related to the main effect of stabilization and time are shown in Table 2. According to the mixed design ANOVA, stabilization was significantly related to the control time. F(4,77) = 2.882, P = 0.028). 

**Table 2:** VAS scores within-subjects effects (time) and between-subjects effects (stabilization and nonstabilization group).

VAS scores (n = 82)	Stabilization Yes, mean ± SD (n = 39)	Stabilization No, mean ± SD (n = 43)	Total	Main effect		Interaction effect	Source of difference for time ****
				Time	Group		
Beginning (1)	7.81 ± 0.76	7.51 ± 0.56	7.67 ± 0.69	V = 0.983* F = 1130.2 47** P < 0.001* **	F = 10.141 P = 0.002	V = 0.130 F = 2.882 P = 0.028	1-2, 1-3, 1-4, 1-5
1st month (2)	4.05 ± 0.62	3.85 ± 0.49	3.95 ± 0.56				
3rd month (3)	3.51 ± 0.51	3.44 ± 0.50	3.47 ± 0.50				
6th month (4)	3.37 ± 0.49	3.18 ± 0.51	3.28 ± 0.50				
12th month (5)	3.28 ± 0.50	2.74 ± 0.50	3.02 ± 0.57				
Total	4.41 ± 1.82	4.14 ± 1.80					
Source of difference for interaction (stabilization × time)							
	Pairwise comparison***** (Time)		P		Pairwise comparison (Time)		P
Stabilization Yes	1-2, 1-3, 1-4, 1-5 2-3, 2-4, 2-5		<0.001	Stabilization No	1-2, 1-3, 1-4, 1-5 2-3, 2-4, 2-5 5-4, 5-3		<0.001
Mauchly’s sphericity test: W = 0.774, χ2 = 20.092, P = 0.017, df = 9, The assumption of sphericity was not met. Multivariate ANOVA (MANOVA) was used.							

*Pillai’s trace test statistical value for MANOVA. **F test statistical value for MANOVA. ***The mean difference is significant at P < 0.05). ****Simple contrasts (first) were used. *****Simple effects analysis with Bonferroni adjustment were used.

A simple effects analysis with a Bonferroni adjustment was used to make a multiple comparison in order to reveal the effect of the meaningful interaction, and the time intervals between the stabilization and nonstabilization groups were compared in pairs. Regarding the source of the difference of interaction (stabilization × time) in Table 2, it can be said that the average VAS scores measured at 1, 3, 6, and 12 months were significantly lower than the average VAS score at the beginning (P < 0.001). Furthermore, the average VAS scores measured at 3, 6, and 12 months were significantly lower than those measured at the 1st month (P < 0.001). 

In the nonstabilized group, the average VAS scores measured at 1, 3, 6, and 

12 months were significantly lower than the average VAS score at the beginning (P < 0.001). In addition, the average VAS scores measured at 3, 6, and 12 months were significantly lower than those measured at the1st month (P < 0.001). Unlike in the stabilized group, the average VAS scores at the 12th month was significantly lower than those at the 3rd and 6th months (p-P < 0.001) (Table 2). 

**Figure 1:**
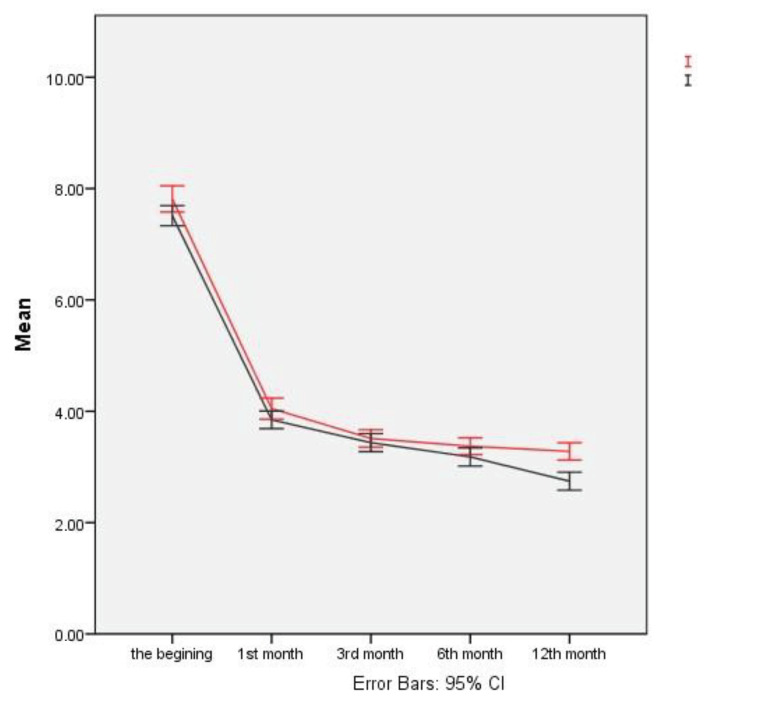
Error bar graph of the stabilization × time interaction for VAS scores.

In addition to the analysis in Table 2, the analytic results are also presented in Figure 1, from which it can be seen that the decreases in VAS scores in the two groups were parallel until the third month, when the decreases in the scores became more significant between the 3rd and 6th months in the nonstabilized group. Furthermore, the decrease in the scores was statistically significant between the 6th and 12th months in the nonstabilized group. 

### 3.3. ODI score results

The average ODI scores decreased when measured every three months during the 12-month period. Changes in the ODI scores measured at the beginning and at 1, 3, 6, and 12 months were assessed with a binary comparison performed by simple context. The mean ODI scores at all times of control were significantly lower than the score recorded at the beginning of the study (P < 0.001). There was a significant main effect of stabilization F(1.80) = 10.141, P = 0.002 that indicates that if we ignored all the other variables, the ODI scores were different for the stabilization and nonstabilization groups. The mean ODI score in the stabilization group (4.41 ± 1.82) was significantly higher than that of the nonstabilization group (4.14 ± 1.80) (P < 0.001). Stabilization had no significant interaction with time F(4.77) = 0.935, P = 0.449. This effect indicates that ODI scores measured at different times were similar for the stabilization and nonstabilization groups (Table 3). 

**Table 3:** ODI scores within-subjects effects (time) and between-subjects effects (stabilization and nonstabilization group).

ODI scores (n = 82)	Stabilization Yes, mean ± SD (n = 39)	Stabilization No, mean ± SD (n = 43)	Total	Main effect		Interaction effect	Source of difference for time
				Time	Group		
Beginning (1)	34.05 ± 1.56	30.74 ± 2.66	32.47 ± 2.71	V = 0.969* F = 604.238* * P < 0.001***	F = 69.479** P < 0.001***	V = 0.046* F = 0.935** P = 0.449	1-2, 1-3, 1-4, 1-5
1st month (2)	24.16 ± 2.67	21.64 ± 2.36	22.96 ± 2.81				
3rd month (3)	23.61 ± 2.35	20.67 ± 1.90	22.20 ± 2.60				
6th month (4)	22.58 ± 1.84	19.97 ± 1.76	21.34 ± 2.18				
12th month (5)	22.16 ± 1.68	19.46 ± 1.67	20.87 ± 2.15				
Total	25.31 ± 4.88	22.50 ± 4.69					
Mauchly’s sphericity test: W = 0.299, χ2 = 94.729, P = 0.001, df = 9. The assumption of sphericity was not met. Multivariate ANOVA (MANOVA) was used.							

*Pillai’s trace test statistical value for MANOVA. **F test statistical value for MANOVA. ***The mean difference is significant at P < 0.05. ****Simple contrasts (first) were used.

**Figure 2:**
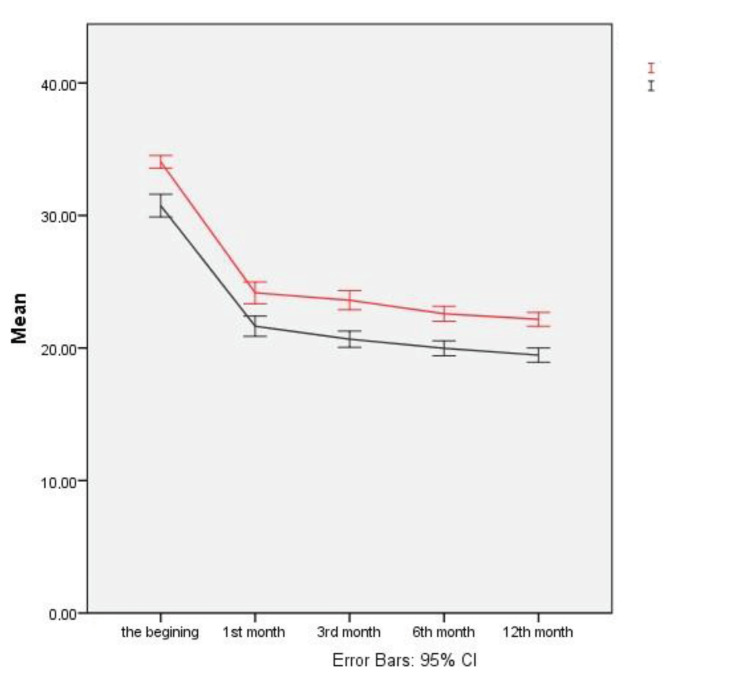
Error bar graph of the stabilization × time interaction for ODI scores.

According to Figure 2, the parallel nature of the graphics of ODI scores of the two groups (the slopes of the red and the black lines are similar) points to the absence of any interaction effect, while the noncrossing confidence intervals in graphs show that there is a statistically significant difference in terms of the main effect of stabilization and time (group and the main effect of time). In addition to the results of the analyses presented in Table 3, the analytical results are also presented in Figure 2. 

All patients were evaluated using a PSS 12 months after the procedure, and overall, 

78.06% of the patients rated the PSS as “very good” or “good”. During the procedure, dural rupture developed in four patients in the stabilization group and in two patients in the nonstabilization group; however, none of these patients developed a spinal headache and no significant permanent complication arose. 

## 4. Discussion 

We aimed to evaluate the efficacy of epiduroscopic treatment in patients with FBSS and with previous stabilized or nonstabilized lumbar surgery in terms of functional quality of life and chronic pain and to evaluate the diagnostic and prognostic value of visual data detected during the procedure. 

In both groups, a significant decrease was observed in the mean VAS scores at all times when compared to the values at the beginning (P < 0.001). 

It was noted that the ODI scores were different for the stabilized and nonstabilized groups, and that stabilization had a significant effect on the ODI score values (P = 0.002). The average ODI score in the stabilized group was significantly higher than that in the nonstabilized group (P < 0.001), and stabilization had no significant interaction with time (P = 0.449). This meant that the ODI scores at different times were similar in the stabilization and nonstabilization groups. When the two groups were compared, the change in the average VAS and ODI scores following mechanical lysis via epiduroscopy was higher in the nonstabilized group. 

Takeshima et al. performed epiduroscopy in patients with FBSS after separating them into groups depending on the presence of nerve roots in the adhesions. They found that epiduroscopic adhesiolysis was an effective treatment in patients with FBSS, and that adhesiolysis of the nerve root may have long-term efficacy in patients who are experiencing pain. They also reported that although the treatment of scar tissue in patients with chronic pain associated with FBSS may improve radicular symptoms, additional factors, such as degeneration in the zygapophysial joint and intervertebral discs and paraspinal muscle spasm may lead to difficulties in returning to daily life (13). 

Unlike the previous epiduroscopic studies, all patients in the present study had FBSS. The FBSS patients were divided into two groups: those who had undergone previous stabilization surgery with instrumentation, and those who had undergone previous lumber disc surgery without instrumentation. The stabilized patients were found to be significantly older in age. Although this may have disrupted the homogeneity of the patient groups, it was consistent with the course of illness, and patients with previous multiple operations would more likely to be older in age than those with a single operation. In our study, we excluded patients with lumbar stenosis and additional disc herniation. Although intense fibrous tissues may lead to difficulties in surgical interventions, we believe that selecting appropriate patients and performing the procedures by an experienced physician have positively affected our outcomes. 

Geurts et al. concluded that epiduroscopy is important in spinal root pathologies as it may identify adhesions that are undetected by MRI, while target-directed epidural drug applications near the spinal nerve may result in serious, long-term pain relief (14). 

Ross et al. (15) reported the severity of fibrosis varies depending on the technique and the number and instrumental properties of the surgery. 

It has been shown that a significant relationship exists between the recurrence grade of scar tissue and radicular pain due to activity (15–18). Fibrinolytic activity defects in FBSS cause adhesion of fibrin and chronic inflammation, and so reducing the fibrotic area in these patients is important (6). Epiduroscopy is known to result in not only mechanical lysis, but also antiinflammatory and pain-relieving effects by washing off the epidural space and administering additional drugs. We think that accurate and detailed information of the patients about the procedure, close postoperative follow-up, and the use of pathological findings obtained from the epidural space for further treatment planning contributed to the decrease in VAS and ODI scores. 

In addition to its mechanical lysis effect on the scar tissue, an epiduroscopy also serves to wash out, dilute, or remove the isotonic and inflammatory agents and chemical and biological mediators through the isotonic solution. Furthermore, the technique allows the application of antiinflammatory drugs directly into the pathological area. An antiinflammatory response is targeted with the medications used during an epiduroscopy (19–21). In particular, steroidal antiinflammatory activity and an epidural washout with saline may suppress the inflammatory mediators that cause pain. As reported in similar studies, the use of local anesthetics with corticosteroids has been shown to have an analgesic and antiinflammatory effect (22–26). 

After observing differences in the ODI values of the groups at the first month, a passive physical therapy program involving home exercise and gabapentin was recommended, especially for the stabilized group. It was observed that the stabilized patients were more agitated about having to return to their active lives, and through this program, we aimed to increase the daytime movement of the patients in the stabilized group who usually had a sedentary life. The presence of widespread and severe fibrotic tissues was an indicator of resistance to epiduroscopic treatment and was one of the key factors in the worsening of outcomes. If needed, the patients were evaluated by physical therapy, neurology and psychiatry clinics, and were given sleeping pills, antidepressants, gabapentin, baclofen-derived drugs, or proper physical therapy programs. No additional invasive procedures were planned. 

During the epiduroscopy, we observed that moderate to severe fibrous tissues were significantly higher in the stabilized patients. Fibrous tissue was found to be moderate in the nonstabilized patients, and the relationship between the severity of fibrosis and the filling defect was evaluated with epiduroscopy. Filling defects were more likely to be observed in the stabilized patients. A reduction in the vascularity of the mature and fibrotic tissues could be distinguished visually, and no vascular structure was identified in some areas. 

Hemmo et al. reported that one of the underlying pathologies associated with FBSS is severe epidural fibrosis, and its prevalence—following detection by epiduroscopy—is considerably high. The incidence of severe fibrosis is higher in patients with histories of wider area surgeries than in patients that underwent noninvasive procedures (9). The study found the sensitivity of epiduroscopy in epidural diagnosis to be 91%, and the ability to detect a pathologic lesion to be 75%. The authors concluded that better outcomes may be achieved and the pathology would be more reachable and improvable in the presence of locally similar pain associated with mild-to-moderate fibrosis. They further declared that patients with severe fibrous tissue, reduction in vascularity, incompatible pain, and filling defects were more resistant to treatment (8). The results of the present study were similar to those of the previous studies in the literature. 

## 4.1. Limitations 

Of the FBSS patients; social, familial, and occupational lifestyles, smoking and alcohol intake habits, and changes in weight during the follow-up period were not recorded. We did not compare the possible changes in VAS and ODI scores that may occur in the case of changing lifestyles during long-term follow-up. Additionally, it would be appropriate to determine the treatment algorithm on a multidisciplinary basis with neurosurgery, neurology, physical therapy, and psychiatry clinics. Also, treatment needs of the patients could be classified according to the reasons. 

## 4.2. Conclusion

In the present study, we found an epiduroscopic adhesiolysis and hyalurinidase-steroid combination to be more effective in the control of pain in patients without stabilization. The lower ratio of benefit in patients with stabilization may be due to the longer periods of illness, larger amounts of scar tissue, accompanying perfusion defects due to the decreased blood circulation caused by fibrous tissue around the nerve, higher incidences of tissue damage during instrumental surgery, the presence of pain memory in these cases, and the presence of a depressive mood in patients who experience chronic pain. These factors may lead to a more difficult recovery process. 

We suggest that an epiduroscopy should be included in the diagnosis and treatment algorithm, particularly of stabilized FBSS patients. Prior to making a resurgery decision or SCI, an epiduroscopy may be considered a useful treatment in experienced centers. We suggest that epiduroscopy may be predictive in prognosis and provides reliable information about the intensity of the scar tissue.
